# An Evaluation of HER2 Expression Heterogeneity in Primary Tumors and Metastatic Lymph Nodes of Patients With Advanced Extramammary Paget's Disease: Prognostic and Therapeutic Implications

**DOI:** 10.1002/cam4.71274

**Published:** 2025-10-07

**Authors:** Zhicheng Liu, Xingliang Tan, Zhiming Wu, Yi Tang, Qianghua Zhou, Wensu Wei, Cong Yang, Long Huang, Yanjun Wang, Kai Yao

**Affiliations:** ^1^ Department of Urology Sun Yat‐sen University Cancer Center Guangzhou China; ^2^ State Key Laboratory of Oncology in Southern China Guangzhou China; ^3^ Collaborative Innovation Center of Cancer Medicine Guangzhou China; ^4^ Guangdong Provincial Clinical Research Center for Cancer Guangzhou China

**Keywords:** antibody–drug conjugate, Extramammary Paget's disease, HER2, metastatic lymph nodes, prognosis

## Abstract

**Background:**

HER2 expression in primary versus metastatic tumors in advanced Extramammary Paget's Disease (EMPD) remains inadequately characterized. This investigation aimed to assess HER2 expression heterogeneity between primary tumors and metastatic lymph nodes (LNs) in patients with advanced EMPD and to assess the prognostic value of HER2 expression and other pathological factors in determining the therapeutic value of HER2 targeting.

**Methods:**

We included 170 patients diagnosed with primary EMPD. Survival outcomes were analyzed using multivariate Cox regression and log‐rank analysis, while inconsistencies in HER2 expression between primary and metastatic LNs were assessed using the kappa coefficient.

**Results:**

HER2 high‐expression was observed in 71.6% of primary tumors and 68.8% of metastatic LNs, with high HER2 expression correlating with poorer overall survival. Multivariate Cox analysis identified advanced N stage and HER2 high‐expression in primary tumors as independent poor prognostic factors. In 31 paired samples, the discordance rate of HER2 status between primary tumors and corresponding LNs was 35.48% (*n* = 11) (Kappa 0.11, 95% CI −0.26 to 0.47; *p* = 0.540), with 19.4% of cases showing a shift from high HER2 expression in primary tumors to low expression in metastatic LNs. Patients treated with disitamab vedotin had an 80% objective response rate (ORR) and a 100% disease control rate (DCR), with no adverse events above grade 3–4.

**Conclusion:**

HER2 was frequently expressed in both primary tumors and metastatic LNs in EMPD patients, though heterogeneity was observed. HER2 status should be assessed in both primary and metastatic sites. Disitamab vedotin shows promise for treating HER2‐positive advanced EMPD, warranting further study.

**Abbreviations**
ADC: antibody–drug conjugates
AE: adverse events
BVI: blood vessel invasion
CEA: carcinoembryonic antigen
CI: confidence interval
CR: complete response
DCR: disease control rate
DTX: docetaxel
EMPD: Extramammary Paget's disease
FP: 5‐fluorouracil + cisplatin
HER2: human epidermal growth factor receptor 2
HR: hazard ratio
IHC: immunohistochemistry
IQR: interquartile range
LNs: lymph nodes
N/A: not applicable
NE: not evaluated
ORR: objective response rate
OS: overall survival
PD: progressive disease
PFS: progression‐free survival
PNI: perineural invasion
PR: partial response
PT: primary tumors
PTX: paclitaxel
S‐1: tegafur
SD: stable disease
SYSUCC: Sun Yat‐sen University Cancer Center

## 1 Introduction

Extramammary Paget's disease (EMPD) is a rare intraepithelial malignant tumor originating from sweat glands and predominantly adenocarcinomatous. In males, it primarily affects the scrotum and penis [1, 2]. While most primary EMPD remains confined to the epidermis, it can invade the dermis, penetrate soft tissues, and metastasize to regional lymph nodes (LNs) and other organs. These patients have advanced or metastatic EMPD, with a poor prognosis and survival rates below 25%. Despite surgical resection, the local recurrence rate reaches 65.2% [3, 4]. Since surgical resection alone is ineffective in such cases, comprehensive treatment is crucial for improving prognosis. No standardized drug regimens for invasive EMPD exist. Docetaxel regimens are common, with a 47.1% effectiveness but high toxicity and side effects [5]. There is an urgent need for novel, more effective, and less toxic pharmacological interventions.

Considering the aforementioned factors, our clinical observations indicate a high prevalence of HER2 among patients in our center. The human epidermal growth factor receptor 2 (HER2), a 185 kilodalton tyrosine kinase receptor, is encoded by the ERBB2 gene located at the 17q12 position on chromosome 17 [6]. HER2 is positively expressed in an estimated 15%–25% of breast cancer cases, 30%–40% of gastric cancer cases, and 40%–50% of uroepithelial cancer cases. The prognosis is poor, as HER2 positivity is linked to increased malignancy. HER2‐targeted therapy is effective in these cancers. Given the similarities between EMPD and these cancers, HER2 might be a crucial treatment target for EMPD patients [7, 8, 9, 10]. Targeted HER2‐ADC therapy may be an effective therapeutic option.

Current studies focus on HER2 in primary EMPD tumors while neglecting paired samples that indicate worse prognosis in patients with lymph node metastases. Investigating HER2 expression in both primary and metastatic sites is crucial for assessing receptor status and supporting HER2‐targeted therapy in EMPD patients with pN+ status. This study explored HER2 expression discrepancies in primary and metastatic EMPD to evaluate the feasibility of targeting HER2 in advanced EMPD.

## 2 Materials and Methods

### 2.1 Patients

Approved by the Sun Yat‐sen University Cancer Center Ethics Committee, this study analyzed 170 patients with scrotal and/or penile EMPD treated at SYSUCC from 2003 to 2023. Clinical data were obtained from patient records. All patients underwent histological evaluation and imaging, including abdominal/pelvic CT, chest X‐rays, cystoscopy, and serum PSA measurements, to exclude Paget's phenomenon from adenocarcinomas of other organs. No specific TNM staging system exists for EMPD, so staging was based on the TNM system by Ohara et al. [11] and clinical guidelines from Kibbi et al. [4]. Of the 170 patients, 90 underwent lymph node dissection, and 50 had pathologically confirmed lymph node metastases. For these patients, only specimens meeting our predefined tissue quality and cellularity criteria were suitable for IHC. Accordingly, we analyzed 102 primary tumor specimens and 32 metastatic lymph node samples (31 paired) for HER2 staining. Unstained or suboptimal specimens were excluded from the HER2 specific analyses but were retained in the survival and multivariate models to preserve the full cohort.

### 2.2 IHC Staining

HER2 expression in EMPD was assessed using IHC with an anti‐HER2/neu (4B5) rabbit monoclonal antibody (Ventana, Roche Diagnostics) on the Ventana BenchMark XT automated staining platform, following the manufacturer's protocol. The HER2 antigen was thermally repaired for 30 min (pH 8.5) and visualized using Hematoxylin II, Bluing Reagent, and the UltraView Universal DAB Detection Kit (all Ventana). All procedures were verified on the Ventana platform, the gold standard for anti‐HER2 therapies [12]. IHC results were evaluated according to 2023 ASCO/CAP guidelines for breast cancer: 0 (no or incomplete faint staining in ≤ 10% of tumor cells), 1+ (faint incomplete staining in > 10%), 2+ (weak to moderate staining in > 10% or strong staining in ≤ 10%), and 3+ (complete, intense staining in > 10% of tumor cells) [13]. An IHC score of 2+ or 3+ indicated HER2 high expression, while 0 or 1+ indicated low expression. FISH results did not impact HER2 expression classification.

### 2.3 Assessment of Metastatic Lymph Node Response and Safety

Metastatic lymph node dimensions were assessed via computed tomography (CT) scans. Treatment efficacy was measured by the Response Evaluation Criteria in Solid Tumors, classifying outcomes into complete response (CR), partial response (PR), stable disease (SD), and progressive disease (PD). The safety analysis of the treatment included the evaluation of adverse effects, which were graded from 1 (mild) to 4 (severe) according to the National Cancer Institute’s Common Terminology Criteria for Adverse Events version 4.0.

### 2.4 Statistical Analysis

Scoring of samples was conducted by a minimum of two pathologists without access to clinical data, ensuring a blind review. Categorical variables were analyzed using the chi‐square test and Fisher’s exact test. Discrepancies in HER2 expression within matched samples were assessed through cross‐tabulation and the calculation of Cohen’s kappa coefficient. Kaplan‐Meier estimation was employed to determine overall survival, defined as the time from the initial resection of the primary EMPD to either death from any cause or the last follow‐up. Progression‐free survival was calculated from the initial resection to disease progression or the last follow‐up. Survival differences were compared using log‐rank tests. Variables significantly affecting survival in univariate analysis were further analyzed using the Cox proportional hazards model for multivariate assessment. Sankey diagrams were utilized to illustrate changes in HER2 expression. Statistical significance was set at a P value less than 0.05. Data analysis was performed using R (version 4.2.1) and Microsoft Excel.

## 3 Result

### 3.1 Patient Characteristics

In this study, we analyzed 170 EMPD patients diagnosed at SYSUCC. The median follow‐up duration was 42.2 months (IQR: 20.3 to 71.7 months). Demographic and clinical characteristics are summarized in Table 1. Ninety patients underwent radical lymph node dissection. The cohort comprised exclusively male patients with primary perineal tumors involving the scrotum and/or penis. Tumor staging indicated that 61 patients had non‐invasive tumors (T0), 52 had superficially invasive tumors within the papillary dermis (T1), and 57 had deeply invasive tumors extending into or beyond the reticular dermis (T2). Adjuvant therapy was administered to 42 (24.7%) patients, encompassing chemotherapy, targeted therapy, and immune therapy, with a subset receiving anti‐HER2 therapy (*n* = 5, 2.9%).

**TABLE 1:** Clinicopathological characteristics in patients with Extramammary Paget's disease.

Variables	*N* (%)
Total (*N* = 170)	
Median follow‐up time (IQR) (months)	42.2 [20.3;71.7]
Median time to progression (IQR) (months)	36.6 [18.4;64.1]
Age at diagnosis (years)	
> 68	78 (45.9%)
≤ 68	92 (54.1%)
Lymph node dissection	
No	80 (47.1%)
Yes	90 (52.9%)
Number of deaths	
No	111 (65.3%)
Yes	59 (34.7%)
Number of progressions	
No	93 (54.7%)
Yes	77 (45.3%)
HER2 IHC scores in primary tumors (PT)	
0	10 (9.8%)
1+	19 (18.6%)
2+	41 (40.2%)
3+	32 (31.4%)
HER2 IHC scores in metastatic lymph nodes (LNs)	
0	7 (21.9%)
1+	3 (9.4%)
2+	9 (28.1%)
3+	13 (40.6%)
HER2 stratification in PT	
Low expression	29 (28.4%)
High expression	73 (71.6%)
HER2 stratification in metastatic LNs	
Low expression	10 (31.3%)
High expression	22 (68.8%)
pT stage^a^	
0/Intraepithelial	61 (35.9%)
1/superficial invasion	52 (30.6%)
2/deep invasion	57 (33.5%)
pN stage	
0	111 (65.3%)
1	12 (7.1%)
2	47 (27.6%)
Pathologic risk factors	
None	130 (76.5%)
BVI	31 (18.2%)
BVI and PNI	9 (5.3%)
Adjuvant therapy	
No	128 (75.3%)
Yes	42 (24.7%)

Abbreviations: BVI, blood vessel invasion; HER2, human epidermal growth factor receptor; IHC, immunohistochemistry; IQR, interquartile range; LNs, lymph nodes; PNI, perineural invasion; PT, primary tumors.

^a^ T0 (in situ), T1 (tumor thickness ≦ 4 mm without lymph vascular invasion), T2 (tumor thickness > 4 mm or lymph vascular invasion), N0 (No LN metastasis), N1 (1 regional LN metastasis), N2 (2 or more regional LN metastases), M0 (no distant or LN metastasis beyond regional LN basin), M1 (distant organ metastasis or LN metastasis beyond LN basin).

### 3.2 IHC Score of HER2 Protein in EMPD Patients

The criteria and expression patterns are shown in Figure S1. Among 102 patients who had HER2 status in their primary tumor sites, HER2 IHC score was 0 in 10 (9.8%), 1+ in 19 (18.6%), 2+ in 41 (40.2%), and 3+ in 32 (31.4%), with a HER2 high‐expression (2+/3+) rate of 71.6%. Table 2 illustrates the correlations between HER2 status and clinical characteristics. No significant differences were noted in the distribution of T and N stages between the primary tumor groups with high HER2 expression and those with low HER2 expression (0/1+). However, age at diagnosis was significantly different between the two groups. HER2 expression was associated with a younger population.

**TABLE 2:** Correlation of HER2 status with clinicopathological parameters in Extramammary Paget's disease of primary lesion.

	Her2‐IHC scores (total%)				*χ* ^2^	*p*	Her‐2 expression		*χ* ^2^	*p* ^a^
	0	1	2	3			High	Low		
	*N* = 10	*N* = 19	*N* = 41	*N* = 32			*N* = 73	*N* = 29		
Age at diagnosis (years)					12.20	0.006^b^			8.79	0.003
> 68	7 (15.6%)	13 (28.9%)	11 (24.4%)	14 (31.1%)			25 (55.6%)	20 (44.4%)		
≤ 68	3 (5.3%)	6 (10.5%)	30 (52.6%)	18 (31.6%)			48 (84.2%)	9 (15.8%)		
*T*					7.71	0.335^b^			2.57	0.277
0	7 (20.0%)	6 (17.1%)	14 (40.0%)	8 (22.9%)			22 (62.9%)	13 (37.1%)		
1	2 (5.6%)	8 (22.2%)	13 (36.1%)	13 (36.1%)			26 (72.2%)	10 (27.8%)		
2	1 (3.2%)	5 (16.1%)	14 (45.2%)	11 (35.5%)			25 (80.6%)	6 (19.4%)		
*N*					5.52	0.564^b^			0.94	0.582^b^
0	8 (12.7%)	11 (17.5%)	23 (36.5%)	21 (33.3%)			44 (69.8%)	19 (30.2%)		
1	0 (0.0%)	3 (37.5%)	2 (25.0%)	3 (37.5%)			5 (62.5%)	3 (37.5%)		
2	2 (6.5%)	5 (16.1%)	16 (51.6%)	8 (25.8%)			24 (77.4%)	7 (22.6%)		
Adjuvant therapy					5.29	0.161^b^			0.00	1.000
No	9 (12.2%)	12 (16.2%)	33 (44.6%)	20 (27.0%)			53 (71.6%)	21 (28.4%)		
Yes	1 (3.6%)	7 (25.0%)	8 (28.6%)	12 (42.9%)			20 (71.4%)	8 (28.6%)		
Pathologic risk factors					2.14	0.961^b^			0.45	0.885^b^
None	8 (11.6%)	13 (18.8%)	27 (39.1%)	21 (30.4%)			48 (69.6%)	21 (30.4%)		
BVI	2 (8.3%)	4 (16.7%)	11 (45.8%)	7 (29.2%)			18 (75.0%)	6 (25.0%)		
BVI and PNI	0 (0.0%)	2 (22.2%)	3 (33.3%)	4 (44.4%)			7 (77.8%)	2 (22.2%)		
Number of deaths					4.49	0.215^b^			1.28	0.257
No	9 (13.4%)	13 (19.4%)	23 (34.3%)	22 (32.8%)			45 (67.2%)	22 (32.8%)		
Yes	1 (2.9%)	6 (17.1%)	18 (51.4%)	10 (28.6%)			28 (80.0%)	7 (20.0%)		
Number of progressions					2.13	0.549^b^			0.07	0.799
No	6 (10.7%)	11 (19.6%)	19 (33.9%)	20 (35.7%)			39 (69.6%)	17 (30.4%)		
Yes	4 (8.7%)	8 (17.4%)	22 (47.8%)	12 (26.1%)			34 (73.9%)	12 (26.1%)		

Abbreviations: BVI, blood vessel invasion; HER‐2, human epidermal growth factor receptor; IHC, immunohistochemistry; PNI, perineural invasion.

^a^ Chi‐square test.

^b^ Fisher's exact test.

In 32 patients who had HER2 status in their metastatic LNs, the HER2 IHC score was 0 in 7 (21.9%), 1+ in 3 (9.4%), 2+ in 9 (28.1%), and 3+ in 13 (40.6%), with a HER2 expression (2+/3+) rate of 68.8%. The correlations between HER2 status and clinical characteristics are shown in Table S1. Similar to those of the primary tumors, no significant disparities were observed between the two groups in terms of pathological characteristics, with the exception of progression status.

Finally, paired samples were collected from 31 patients with primary tumors and corresponding metastases, and HER2 expression is shown in Table S2. In the paired samples, the rates of HER2 high expression were 74.5% (23/31) and 71.0% (22/31) in primary and metastatic LNs, respectively. HER2 high expression in metastases, as observed in primary tumors, appears to be strongly associated with younger patients (< 68).

### 3.3 Change in HER2 Status Between Primary Site and Metastatic LNs

**FIGURE 1:**
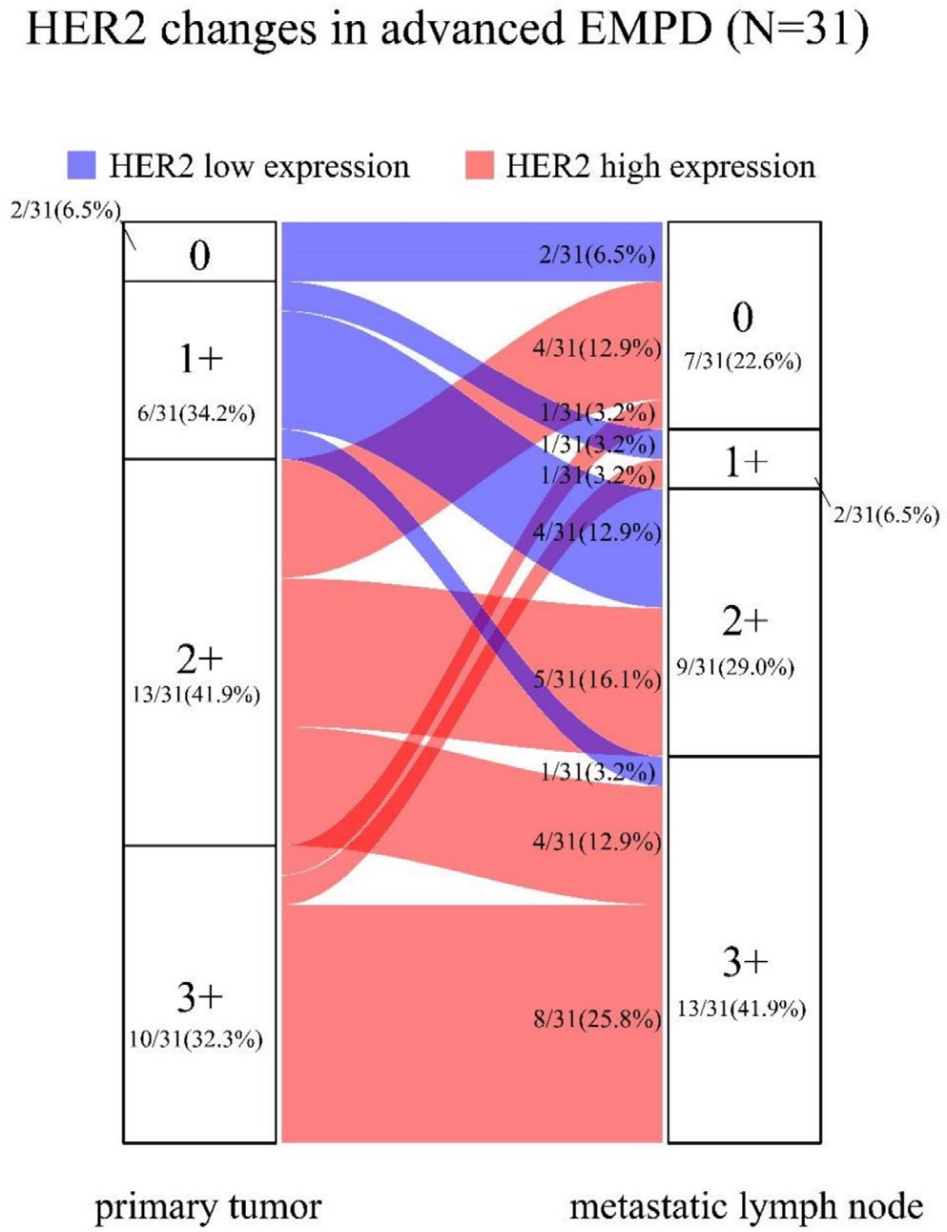
Expression changes of HER2 between primary and metastatic lymph nodes in EMPD patients.

The Sankey diagram highlights discrepancies in HER2 expression between primary tumors and metastases in the 31 paired patients (Figure 1). Only 16 out of 31 patients (51.61%) had matching primary and metastatic HER2 scores. After grouping them based on whether HER2 was highly expressed, a 35.48% (95% CI, 17.64%–53.32%) (*n* = 11) discrepancy in HER2 status was noted between the primary tumor and the matched metastatic LNs (Kappa = 0.11, 95% CI, −0.26 to 0.47, *p* = 0.540). In 20 out of 31 patients (64.52%), there was concordance between the expression levels of primary and metastatic LNs; in 6 out of 31 (19.35%) patients, the HER2 expression at primary sites was high, but that at metastatic LNs was low; and in 5 out of 31 (16.13%) patients, the HER2 expression at metastatic LNs was low, but that at primary sites was high (Table 3).

**TABLE 3:** Concordance of immunohistochemical (IHC) results for HER2 protein between primary and metastatic sites.

Metastatic LNs	Primary lesion		*χ* ^2^	*p* ^a^	Total	Kappa (95% CI)	Concordance (95% CI)	*p*
	High expression	Low expression						
High expression	17 (77.3%)	5 (22.7%)	0.03	0.660	22	0.11 (−0.26, 0.47)	64.52% (45.37%–80.77%)	0.540
Low expression	6 (66.7%)	3 (33.3%)			9			
Total	23	8			31			

Abbreviations: HER2, human epidermal growth factor receptor; IHC, immunohistochemistry; LNs: lymph nodes.

^a^ Fisher's exact test.

### 3.4 Survival Analysis

**FIGURE 2:**
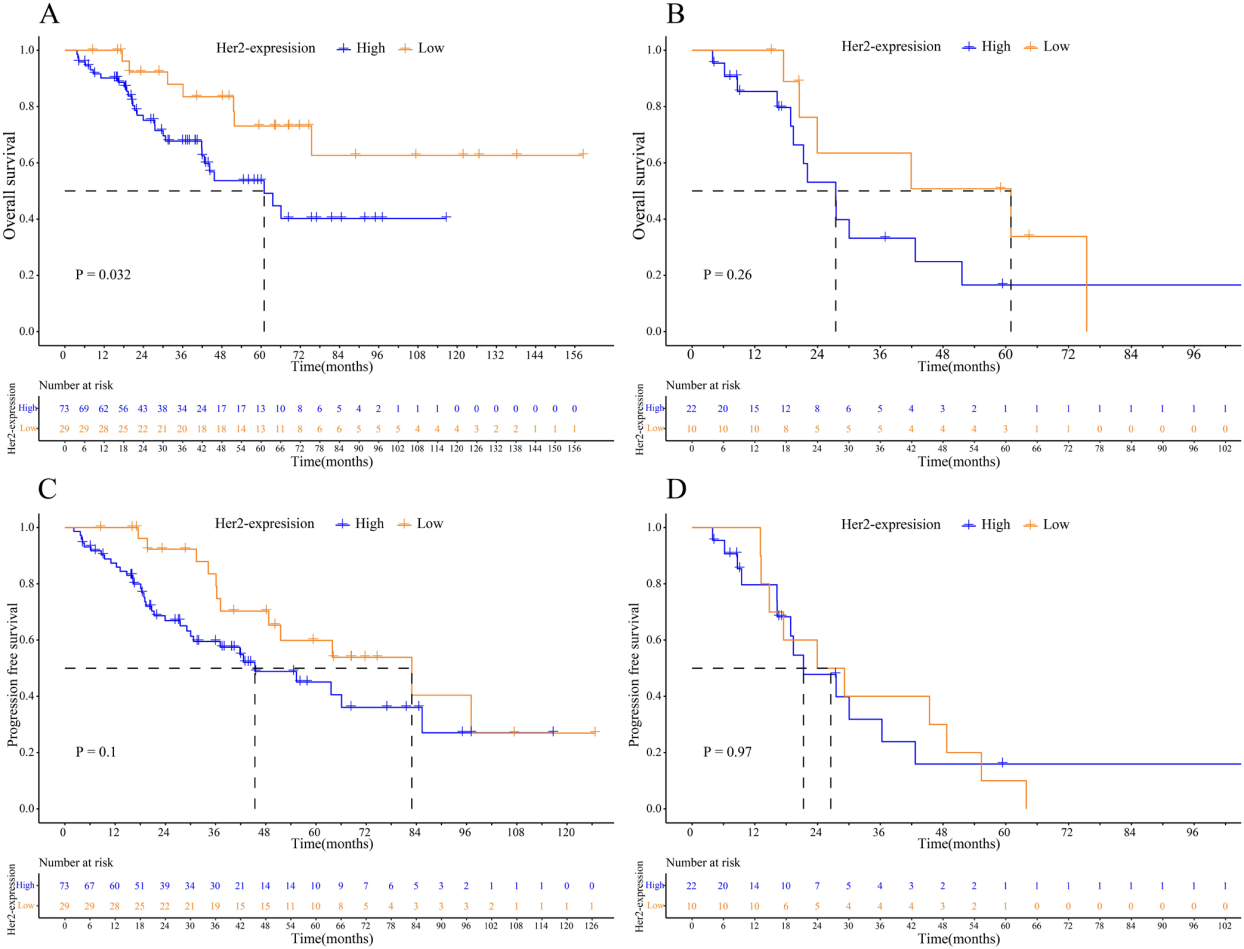
Survival analyses of EMPD patients with different HER2 expression. (A, C) Kaplan–Meier survival curves by log‐rank test were performed to explore the difference of OS and PFS between HER2 high or low expression in primary tumors. (B, D) Kaplan–Meier survival curves by log‐rank test were performed to explore the difference of OS and PFS between HER2 high or low expression in metastatic LNs. EMPD, Extramammary Paget's disease; LNs, lymph nodes; OS, overall survival; PFS, progression‐free survival.

The correlations between *T* and *N* stages and patient survival are shown in Figure S2, which shows good predictability for both OS and RFS. Notably, N staging has emerged as a crucial factor in predicting survival prognosis. Overall, the survival analysis indicated that patients with HER2 high‐expression in the primary tumor had a significantly shorter OS than those with low expression, and the remaining endpoints did not show statistical differences (Figure 2). Also, short‐term PFS was better in the HER2‐low group while the *p*‐value is 0.1 (Figure 2C). To account for the effect of N stage and other factors on prognosis, we performed univariate Cox analysis (Table 4), and those that were significant were further included in a multivariate Cox analysis, which showed that in addition to N stage, HER2 high‐expression in the primary tumors was also a detrimental factor after adjustment (Table 4). Additional survival analyses for paired patients revealed an interesting phenomenon: when patients had HER2 high‐expression in both primary and metastatic LNs, their prognosis was significantly worse prognosis compared to other groups, particularly in terms of OS. For reference, the survival curves are displayed in Figure S3.

**TABLE 4:** Uni‐ and multivariate Cox regression model of pathological features for the prediction outcomes in Extramammary Paget's Disease patients.

Variables	Univariate Cox				Multivariate Cox			
	PFS		OS		PFS		OS	
	HR (95% CI)	*p*	HR (95% CI)	*p*	HR (95% CI)	*p*	HR (95% CI)	*p*
HER2 expression								
High versus low	1.73 (0.89–3.37)	0.105	2.42 (1.05–5.60)	0.038	1.63 (0.61–4.00)	0.284	3.68 (1.49–9.09)	0.005
Lymph node dissection								
Yes versus no	1.72 (1.08–2.72)	0.212	1.67 (0.99–2.82)	0.057	0.77 (0.25–2.43)	0.661	0.29 (0.09–0.92)	0.036
pT stage								
T1/2 versus T0	1.99 (1.20–3.31)	0.008	2.56 (1.38–4.78)	0.003	1.00 (0.38–2.65)	0.997	1.22 (0.44–3.35)	0.700
pN stage								
*N*+ versus N0	5.91 (3.62–9.65)	< 0.001	7.59 (4.23–13.60)	< 0.001	1.24 (0.28–5.48)	0.775	15.39 (3.79–62.43)	< 0.001
Pathologic risk factors								
BVI versus none	5.42 (3.30–8.91)	< 0.001	7.14 (4.04–12.60)	< 0.001	7.15 (1.86–27.39)	0.004	2.23 (0.87–5.69)	0.095

*Note:* All variables were transformed into categorical variables. We selected variables using the enter approach. The *p* value threshold was 0.1 (*p* > 0.1) for the removal of insignificant variables from the model. Only variables significantly associated with survival were included in the further analysis.

Abbreviations: BVI, blood vessel invasion; CI, confidence interval; HER2, human epidermal growth factor receptor; HR, hazard ratio; OS, overall survival; PFS, progression‐free survival.

### 3.5 Example of an Application

Table S3 presents efficacy outcomes for five advanced EMPD patients treated with disitamab vedotin (RC48), a HER2‐targeting antibody‐drug conjugate (ADC) conjugated to monomethyl auristatin E (MMAE). None of the patients achieved a complete response (CR). Of the 5 patients with a confirmed response, 4 (80%) had a PR, and 1 (20%) had an SD. The objective response rate (ORR) was 80%, and the disease control rate (DCR: CR + PR + SD) was 100%. At the conclusion of the study, all patients were still alive. The most frequent hematologic side effect was anemia, experienced by three patients (60%) at grade 1 during the treatment period. All patients experienced grade 2 alopecia, four had gastrointestinal disturbances, two experienced peripheral neuropathy in their extremities, and two patients developed localized rashes. All adverse events were reversible, and no patients experienced grade 3 or higher AEs. Figure S4 compares CT images of metastatic LNs in two patients before and after treatment with RC48, confirming a favorable outcome. In contrast, previous chemotherapy regimens were generally ineffective in lymph node metastasis‐positive sites. The current RC48 regimen has satisfactory efficacy and significantly lower toxicity compared to previous regimens in terms of both efficacy and safety (Table S4).

## 4 Discussion

EMPD is characterized by a high rate of surgical recurrence and a low survival rate upon metastasis, underscoring the need for multimodal therapeutic approaches. Yet, no effective chemotherapy protocols have been established for advanced EMPD, especially in patients with extensive lymph node metastases or when the tumor is unresectable. Previous studies have shown that HER2 is highly expressed in EMPD and promotes tumor progression in various cancers. Encouragingly, recent studies have reported that good responses to anti‐HER2 therapy have been observed in advanced EMPD patients [14, 15, 16, 17], indicating the potential of HER2‐targeted treatments for patients with high HER2 expression. However, the literature has mainly examined HER2 expression in primary EMPD tumors, neglecting its expression in metastatic lymph nodes, which is critical given the poorer prognosis associated with such metastases. Moreover, conventional chemotherapy has generally been ineffective in treating lymph node metastases. To address this gap and explore the therapeutic potential of anti‐HER2 treatments in advanced EMPD patients with lymph node involvement, our study undertook the following innovative work.

First, we further explored the relationship between HER2 expression in primary tumors and pathological factors to better guide the treatment of EMPD patients. Some studies have recently reported 15%–60% overexpression of HER2 in primary EMPD. In our study, we reported a HER2 high‐expression rate of 71.6%, which is higher than that reported in a previous study (60%) [1, 18]. The greater number of invasive EMPD patients at our center may explain this finding. This finding aligns with Richter et al.'s study [19], which reported higher IHC HER2 scores (2+ or 3+) in invasive Paget's disease patients than in noninvasive patients (71% vs. 54%). After in‐depth analysis of the relationship between pathological factors and HER2 expression, our study revealed that HER2 high‐expression was an independent poor prognostic factor, further demonstrating the necessity of HER2 as a therapeutic target.

Second, all eligible patients underwent complete lymph node dissection, resulting in comprehensive and detailed data. To the best of our knowledge, this is the first study to thoroughly examine metastatic lymph node data. Previous EMPD studies have described only the HER2 status at primary tumors and corresponding metastatic lesions in one Japanese study, which included diverse metastatic sites. This was a single‐center study of lymph nodes with the largest sample size, which can contribute to an in‐depth investigation of the relationship between primary and metastatic LNs and HER2 expression status in advanced EMPD patients. The detailed clearance data can also contribute to better TNM staging, which may help to better predict patient prognosis. These findings indicate that HER2 expression in the primary tumor does not necessarily impact the ability of tumor cells in metastatic LNs to express the same HER2 levels. Furthermore, HER2‐targeted therapy may not benefit some patients even if their primary tumors are HER2‐positive, while conversely, some patients may benefit from HER2‐targeted therapy even if their primary tumors are HER2‐negative. Therefore, screening for HER2 status in patients with advanced EMPD and lymph node metastases in both primary and metastatic LNs is recommended whenever possible.

Third, we analyzed HER2 discordance in 31 paired primary–metastatic samples. The overall discordance rate was 35.48%. It revealed an inconsistency in the rate of HER2 status between advanced EMPD patients and patients with lymph node metastases. The most common change was a switch from HER2 high‐expression to HER2 low‐expression. Additionally, patients with high HER2 expression in both primary tumors and metastases had a poorer prognosis. This suggests that more aggressive anti‐HER2 therapy may be necessary for this group of patients. For patients with high expression of HER2 in both primary and metastatic LNs (17/31, 54.8%), we recommend anti‐HER2 therapy. For patients with HER2 high‐expression primary tumors and low‐expression in metastatic LNs (5 out of 31, 16.1%), other treatment options may need to be explored. However, in the case of breast cancer, it is common for the HER2 status to evolve. Due to the effectiveness of the ADC anti‐HER2 drug, the transition from high expression in the primary cancer to low expression in the metastases did not strongly affect the continuation of anti‐HER2 targeted therapy [20, 21, 22].

Inconsistency in HER2 expression between primary and metastatic LNs was also reported by Tanaka et al., who reported an inconsistency rate of 8% in 26 patients [23]. Similarly, in a systematic review and meta‐analysis, discordance in HER2 expression between primary and metastatic tumors has been reported in breast cancer, with an inconsistency rates of 5.5% (3.6%–8.5%) and 43.2% (35.2%–51.3%), respectively [24, 25]. This discrepancy may be due to several factors. First, our focus was on metastatic lymph node samples, whereas other studies did not have the same metastatic sites, and distant metastases made up the majority of their samples rather than adjacent lymph nodes. Second, due to the small number of patients with this disease, samples are often collected over a wide span of years. Additionally, the quality of the paraffin‐embedded specimens can affect protein degradation, and differences in technical level (including HER2 testing) among various centers may lead to varying expression scores. Ultimately, the observed inconsistency could be attributed to intratumor heterogeneity in HER2 amplification. During tumor progression, genetic variations or the selection of specific HER2 phenotypes may lead to such discrepancies.

Indeed, we can note that the overall HER2 high/low expression rates of metastases and the primary site were similar in this study, with no significant difference in the chi‐square distribution. However, an internal shift from low to high or high to low expression was observed, resulting in a 35.48% inconsistency rate. Collectively, this evidence shows that HER2 expression can vary as the tumor progresses, necessitating assessment in metastatic LNs for EMPD patients being considered for anti‐HER2 therapy. After testing the primary tumors and metastases for HER2, we can determine a more precise prognosis for patients and effectively guide subsequent individualized anti‐HER2 treatment.

Finally, we have recently gathered compelling efficacy data on ADC therapy. In our center, all five patients treated with RC48 (Table S3) have shown significant therapeutic responses, with four patients achieving partial response (PR) and one experiencing stable disease (SD), resulting in an objective response rate of 80%. Notably, no grade 3 or higher adverse events (AEs) were observed. Despite these promising results, there is currently no standardized treatment protocol for advanced EMPD. Traditional chemotherapy regimens, such as single‐agent paclitaxel/docetaxel and taxane/platinum‐based drugs, have been widely reported but exhibit variable efficacy due to patient heterogeneity [5, 26, 27, 28, 29, 30, 31, 32]. Moreover, these conventional chemotherapy protocols are also associated with a high incidence of grade 3 or 4 myelosuppression [29, 31, 32, 33]. For instance, docetaxel, despite being the most commonly used agent, has an efficacy rate of only 47.1% [5] and is accompanied by significant toxicity and side effects. These limitations underscore the urgent need for more effective, less toxic treatment options. Beyond RC48, emerging ADCs such as enfortumab vedotin (targeting Nectin‐4) and trastuzumab deruxtecan (HER2‐targeted) have also been explored in EMPD and have shown promising therapeutic potential [34, 35]. The positive outcomes of HER2‐ADC targeted therapy not only provide a new therapeutic option but also suggest a promising future in the treatment of advanced EMPD.

This study faced several constraints. First, the small sample size was a limitation due to the disease's low prevalence. Second, this retrospective analysis carries inherent risks of selection bias due to its observational design. Furthermore, findings may have limited generalizability as our cohort exclusively comprised Asian patients. To confirm the results, future research should involve larger, multi‐institutional studies or prospective trials. Also, the data used were from 2003 to 2023. Although our protocols reduced pre‐analytical variability, future studies should use fresh specimens to rule out storage time effects. Third, there was a selection bias in treatment. Advanced EMPD patients received various chemotherapy or targeted therapy regimens, and the use of adjuvant therapies was not uniform across the study period. Additionally, a minority of HER2‐positive EMPD patients were treated with HER2‐targeted therapies in this analysis. Fourth, our study revealed low survival rates in lymph node‐positive patients. Subgroup survival analyses indicated that N stage was the main factor affecting survival in patients with this disease. Therefore, we did not present additional survival curves but instead attempted to correct for this effect using multifactorial Cox regression. Finally, our study did not provide any information on HER2 protein‐encoding gene amplification due to the limited technology available in the early years, the small number of patients, and the fact that clinicians do not routinely prescribe the FISH test, and we plan to include FISH test results as much as possible in future studies.

## 5 Conclusion

To summarize, our study revealed that the overall HER2 high‐expression rate among EMPD patients was 71.6%. In cases involving lymph node metastases, HER2 high‐expression was observed in 74.2% of primary tumors and 71.0% of metastatic lymph nodes. The HER2 status showed some heterogeneity between primary tumors and corresponding metastatic lymph nodes. To more accurately determine the prognosis of patients receiving HER2‐targeted therapy, we recommend assessing HER2 status at both primary and metastatic sites whenever possible. Aggressive anti‐HER2 therapy is recommended for patients with advanced EMPD who have both primary and metastatic LNs with HER2 high‐expression. A positive lymph node status and HER2 high‐expression are poor prognostic factors in patients with EMPD. RC48 demonstrated significant efficacy in treating HER2 high‐expression advanced EMPD patients and has promising potential that requires further validation.

## Author Contributions

**Zhicheng Liu:** conceptualization, investigation, funding acquisition, writing – original draft, methodology, data curation, formal analysis, project administration, validation, visualization, writing – review and editing, software, supervision, resources. **Xingliang Tan:** conceptualization, investigation, funding acquisition, methodology, validation, visualization, writing – review and editing, writing – original draft. **Zhiming Wu:** funding acquisition, resources, validation, conceptualization, investigation; writing – original draft, writing – review and editing, data curation. **Yi Tang:** funding acquisition, resources, validation, visualization, project administration, supervision. **Qianghua Zhou:** methodology, software, visualization, resources, validation, investigation, project administration. **Wensu Wei:** investigation, validation, supervision, funding acquisition, project administration. **Cong Yang:** methodology, visualization, investigation, data curation. **Long Huang:** software, visualization, methodology. **Yanjun Wang:** methodology, validation, visualization, project administration, formal analysis, software, funding acquisition. **Kai Yao:** funding acquisition, visualization, validation, writing – review and editing, project administration, resources, supervision.

## Ethics Statement

Research approval was obtained from the SYSUCC Ethics Committee (SL‐B2024‐048‐01).

## Consent

Informed consent for the clinical details was obtained from EMPD patients (SL‐B2024‐048‐01). All of the authors agreed with the content of the paper and agreed to publish it.

## Conflicts of Interest

The authors declare no conflicts of interest.

## Supporting information


**Figure S1:** The IHC expression pattern of HER2 in EMPD.
**Figure S2:** Survival analyses of EMPD patients with different TN stages.
**Figure S3:** Survival analyses of paired EMPD patients between HER2 status changes.
**Figure S4:** CT image of the metastatic LNs.
**Table S1:** Correlation of HER2 status with clinicopathological parameters in metastatic LNs from patients with Extramammary Paget's disease.
**Table S2:** Correlations of HER2 status with clinicopathological parameters in patients with EMPD according to paired data.
**Table S3:** Efficacy outcomes of 5 patients with Extramammary Paget's disease treated with RC48 at our center.
**Table S4:** Comparison of the current study and previous studies on the efficacy and safety of different regimens in patients with advanced EMPD.

## Data Availability

The datasets supporting the conclusions of this article are available from the corresponding author upon reasonable request. The authenticity of this article has been validated by uploading the key raw data onto the Research Data Deposit platform RDDA2024783239 (www.researchdata.org.cn).
