# Supplementary material for: An Evaluation of HER2 Expression Heterogeneity in Primary Tumors and Metastatic Lymph Nodes of Patients With Advanced Extramammary Paget's Disease: Prognostic and Therapeutic Implications
Source: Cancer Med. 2025 Oct 7;14(19):e71274. doi: 10.1002/cam4.71274 (PMC12501768; doi:10.1002/cam4.71274)
Supplement: Supplementary file 1 — Figure S1: The IHC expression pattern of HER2 in EMPD. Figure S2: Survival analyses of EMPD patients with different TN stages. Figure S3: Survival analyses of paired EMPD patients between HER2 status changes. Figure S4: CT image of the metastatic LNs. Table S1: Correlation of HER2 status with clinicopathological parameters in metastatic LNs from patients with Extramammary Paget's disease. Table S2: Correlations of HER2 status with clinicopathological parameters in patients with EMPD according to paired data. Table S3: Efficacy outcomes of 5 patients with Extramammary Paget's disease treated with RC48 at our center. Table S4: Comparison of the current study and previous studies on the efficacy and safety of different regimens in patients with advanced EMPD. [file CAM4-14-e71274-s001.docx]

**An Evaluation of HER2 Expression Heterogeneity in Primary Tumors and Metastatic Lymph Nodes of Patients with Advanced Extramammary Paget's Disease: Prognostic and Therapeutic Implications**

**Supplementary Materials**

**Supplementary Figures**

**Figure. S1 The IHC expression pattern of HER2 in EMPD.**

IHC was performed to measure the expression of HER2 in EMPD. IHC scores of 0 and 1+ were regarded as low HER2 expression, and scores of 2+ or 3+ were regarded as high HER2 expression (magnification: 100x (left), 400x (right); scale bar, 100 μm). EMPD: Extramammary Paget's disease; HER2: human epidermal growth factor receptor 2; IHC: immunohistochemistry.

**
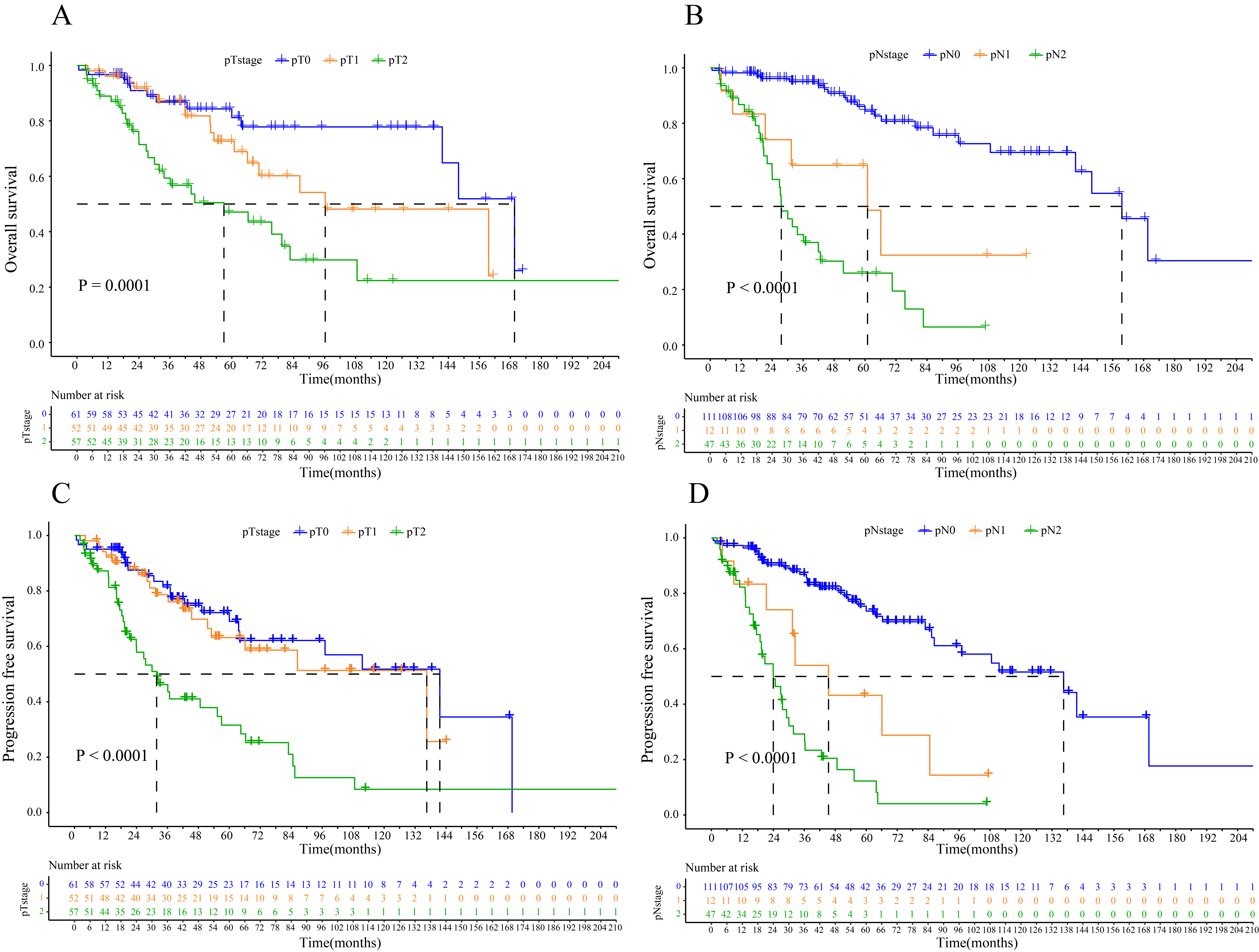
**

**Figure. S2** **Survival analyses of EMPD patients with different TN stages.**

(A, C) Kaplan-Meier survival curves by log-rank test were performed to explore the difference of OS and PFS in different T stages. (B, D) Kaplan-Meier survival curves by log-rank test were performed to explore the difference of OS and PFS in different N stages. EMPD: Extramammary Paget's disease; OS: overall survival; PFS: progression-free survival.

**
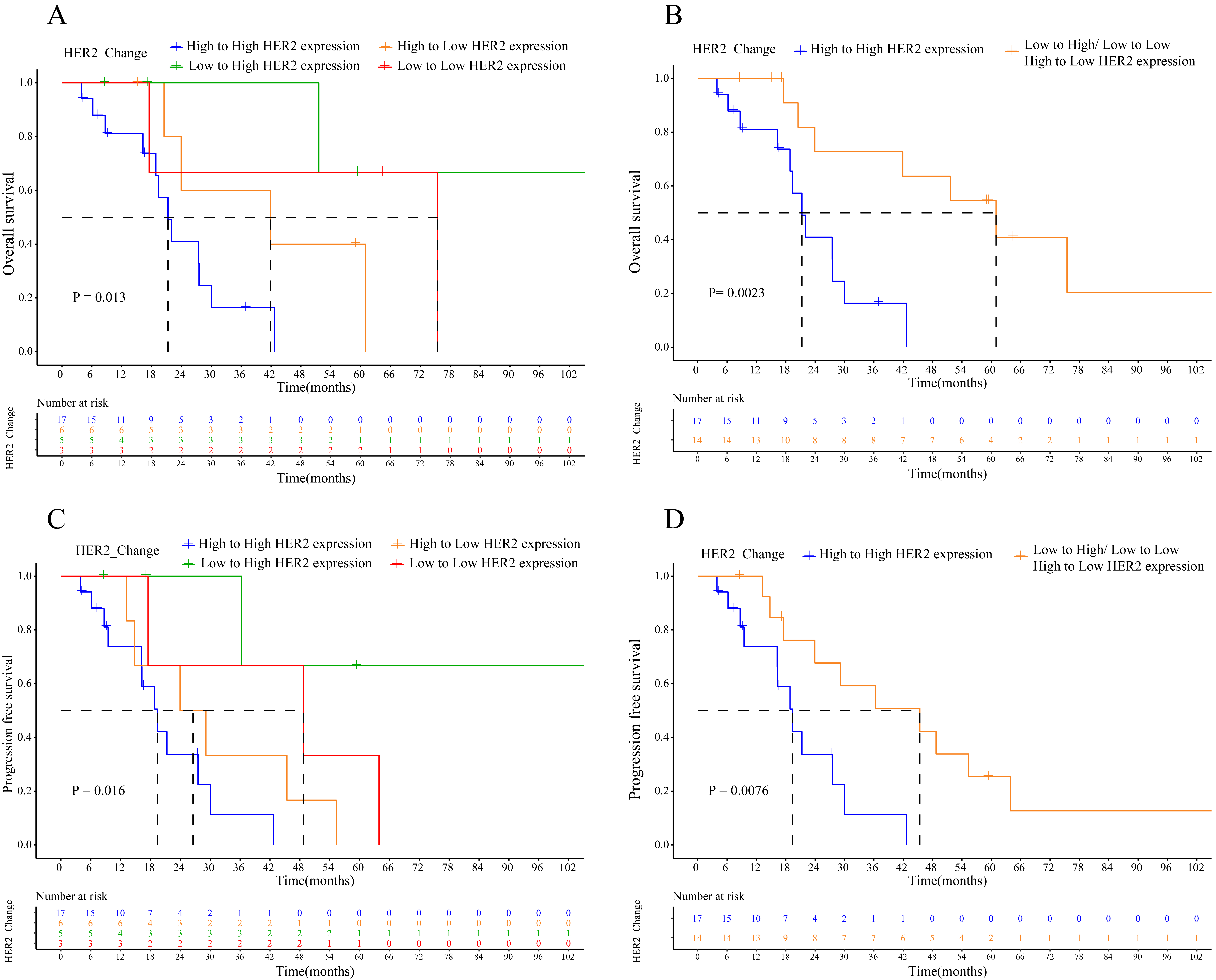
**

**Figure. S3** **Survival analyses of paired EMPD patients between HER2 status changes.**

(A, C) Kaplan-Meier survival curves by log-rank test were performed to explore the difference of OS and PFS among 4 trends of groups. (B, D) Kaplan-Meier survival curves by log-rank test were performed to explore the difference of OS and PFS in 2 trends of groups. EMPD: Extramammary Paget's disease; OS: overall survival; PFS: progression-free survival


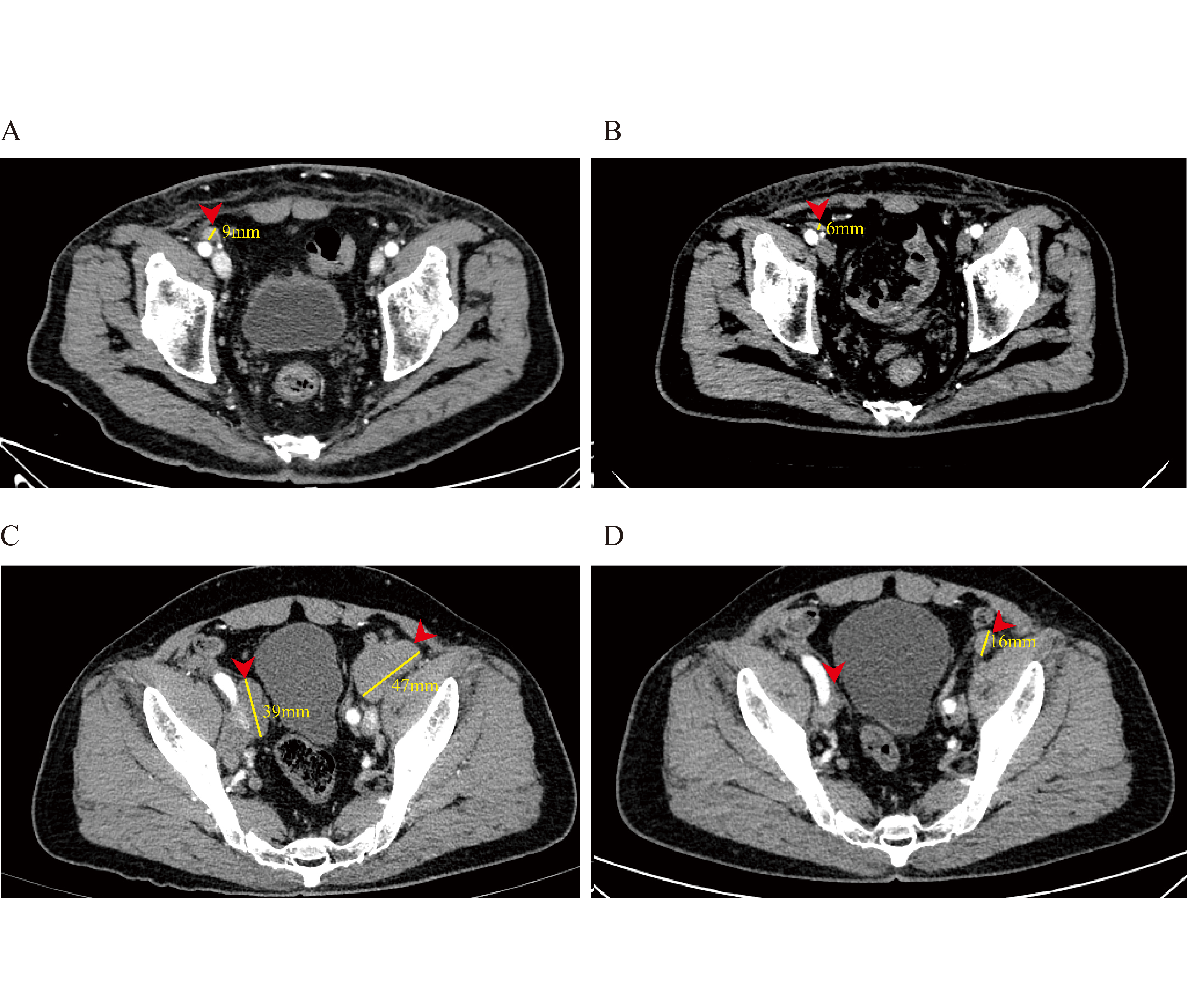


**Figure. S4**: **CT image of the metastatic LNs.**

(A) Pretreatment CT images of patient #3, which demonstrate multiple lymph node metastases in the bilateral iliac vessels. The largest metastasis has a short diameter measurement of approximately 9 mm(arrow); (B) Post-treatment CT images of patient #3, following the RC48 infusion, showing decrease in the largest metastasis to 6 mm with complete resolution of all other measurable disease. (C) Pretreatment CT images of patient #2, which demonstrate multiple lymph node metastases in the bilateral iliac vessels. The largest metastasis measures approximately 39*19mm on the right and 47*26mm on the left(arrow); (D) Post-treatment CT images of patient #2, following the RC48 infusion, showing decrease in the left metastasis to 16*12 mm with complete resolution in the right.

**Supplementary Tables**

**Table. S1** Correlation of HER2 status with clinicopathological parameters in metastatic LNs from patients with Extramammary Paget’s disease.

|  | **Her2-IHC scores(total%)** | | | | |  |  | **Her-2 expression** | |  |  |
| --- | --- | --- | --- | --- | --- | --- | --- | --- | --- | --- | --- |
|  | **0**  **N=7** | | **1**  **N=3** | **2**  **N=9** | **3**  **N=13** | **χ2** | **P-value^b^** | **High**  **N=22** | **Low**  **N=10** | **χ2** | **P-value^a^** |
| **Age at diagnosis (years):** |  | |  |  |  | 3.54 | 0.362 |  |  | 1.92 | 0.128^b^ |
| >68 | 5 (33.3%) | | 2 (13.3%) | 4 (26.7%) | 4 (26.7%) |  |  | 8 (53.3%) | 7 (46.7%) |  |  |
| ≤68 | 2 (11.8%) | | 1 (5.9%) | 5 (29.4%) | 9 (52.9%) |  |  | 14 (82.4%) | 3 (17.6%) |  |  |
| **T:** |  | |  |  |  | 3.64 | 0.776 |  |  | 0.04 | 1.000 |
| 0 | 1 (33.3%) | | 0 (0.0%) | 2 (66.7%) | 0 (0.0%) |  |  | 2 (66.7%) | 1 (33.3%) |  |  |
| 1 | 2 (22.2%) | | 1 (11.1%) | 2 (22.2%) | 4 (44.4%) |  |  | 6 (66.7%) | 3 (33.3%) |  |  |
| 2 | 4 (20.0%) | | 2 (10.0%) | 5 (25.0%) | 9 (45.0%) |  |  | 14 (70.0%) | 6 (30.0%) |  |  |
| **N:** |  | |  |  |  | 2.70 | 0.476 |  |  | 0.00 | 1.000^b^ |
| 1 | 0 (0.0%) | | 1 (20.0%) | 1 (20.0%) | 3 (60.0%) |  |  | 4 (80.0%) | 1 (20.0%) |  |  |
| 2 | 7 (25.9%) | | 2 (7.4%) | 8 (29.6%) | 10 (37.0%) |  |  | 18 (66.7%) | 9 (33.3%) |  |  |
| **Adjuvant therapy:** |  | |  |  |  | 3.22 | 0.472 |  |  | 0.82 | 0.265 |
| NO | 3 (20.0%) | | 0 (0.0%) | 5 (33.3%) | 7 (46.7%) |  |  | 12 (80.0%) | 3 (20.0%) |  |  |
| YES | 4 (23.5%) | | 3 (17.6%) | 4 (23.5%) | 6 (35.3%) |  |  | 10 (58.8%) | 7 (41.2%) |  |  |
| **Pathologic risk factors:** | |  |  |  |  | 9.14 | 0.294 |  |  | 0.22 | 1.000^b^ |
| None | | 0 (0.0%) | 2 (40.0%) | 2 (40.0%) | 1 (20.0%) |  |  | 3 (60.0%) | 2 (40.0%) |  |  |
| BVI | | 5 (25.0%) | 1 (5.0%) | 6 (30.0%) | 8 (40.0%) |  |  | 14 (70.0%) | 6 (30.0%) |  |  |
| BVI and PNI | | 2 (28.6%) | 0 (0.0%) | 1 (14.3%) | 4 (57.1%) |  |  | 5 (71.4%) | 2 (28.6%) |  |  |
| **Number of death:** |  | |  |  |  | 1.44 | 0.744 |  |  | 0.00 | 1.00^b^ |
| NO | 3 (23.1%) | | 1 (7.7%) | 5 (38.5%) | 4 (30.8%) |  |  | 9 (69.2%) | 4 (30.8%) |  |  |
| YES | 4 (21.1%) | | 2 (10.5%) | 4 (21.1%) | 9 (47.4%) |  |  | 13 (68.4%) | 6 (31.6%) |  |  |
| **Number of progression:** |  | |  |  |  | 7.31 | 0.072 |  |  | 3.85 | 0.030^b^ |
| NO | 0 (0.0%) | | 0 (0.0%) | 5 (55.6%) | 4 (44.4%) |  |  | 9 (100.0%) | 0 (0.0%) |  |  |
| YES | 7 (30.4%) | | 3 (13.0%) | 4 (17.4%) | 9 (39.1%) |  |  | 13 (56.5%) | 10 (43.5%) |  |  |

^a^Chi-square test; ^b^Fisher's exact test; BVI, blood vessel invasion; HER-2, human epidermal growth factor receptor; IHC, immunohistochemistry; PNI, perineural invasion;

**Table. S2** Correlations of HER2 status with clinicopathological parameters in patients with EMPD according to paired data.

|  | **Her-2 expression in primary** | |  |  | **Her-2 expression in metastatic** | |  |  |
| --- | --- | --- | --- | --- | --- | --- | --- | --- |
|  | **High**  **N=23** | **Low**  **N=8** | **χ2** | **P-value^b^** | **High**  **N=22** | **Low**  **N=9** | **χ2** | **P-value^b^** |
| **Age at diagnosis (years):** |  |  | 0.00 | 1.000 |  |  | 2.88 | 0.054 |
| >68 | 11 (73.3%) | 4 (26.7%) |  |  | 8 (53.3%) | 7 (46.7%) |  |  |
| ≤68 | 12 (75.0%) | 4 (25.0%) |  |  | 14 (87.5%) | 2 (12.5%) |  |  |
| **T:** |  |  | 3.88 | 0.118 |  |  | 0.18 | 1.000 |
| 0 | 1 (33.3%) | 2 (66.7%) |  |  | 2 (66.7%) | 1 (33.3%) |  |  |
| 1 | 6 (66.7%) | 3 (33.3%) |  |  | 6 (66.7%) | 3 (33.3%) |  |  |
| 2 | 16 (84.2%) | 3 (15.8%) |  |  | 14 (73.7%) | 5 (26.3%) |  |  |
| **N:** |  |  | 0.06 | 0.583 |  |  | 0.00 | 1.000 |
| 1 | 3 (60.0%) | 2 (40.0%) |  |  | 4 (80.0%) | 1 (20.0%) |  |  |
| 2 | 20 (76.9%) | 6 (23.1%) |  |  | 18 (69.2%) | 8 (30.8%) |  |  |
| **Adjuvant therapy:** |  |  | 0.00 | 1.000 |  |  | 0.46 | 0.433 |
| NO | 11 (73.3%) | 4 (26.7%) |  |  | 12 (80.0%) | 3 (20.0%) |  |  |
| YES | 12 (75.0%) | 4 (25.0%) |  |  | 10 (62.5%) | 6 (37.5%) |  |  |
| **Pathologic risk factors:** |  |  | 1.72 | 0.508 |  |  | 0.04 | 1.000 |
| None | 2 (50.0%) | 2 (50.0%) |  |  | 3 (75.0%) | 1 (25.0%) |  |  |
| BVI | 15 (75.0%) | 5 (25.0%) |  |  | 14 (70.0%) | 6 (30.0%) |  |  |
| BVI and PNI | 6 (85.7%) | 1 (14.3%) |  |  | 5 (71.4%) | 2 (28.6%) |  |  |
| **Number of deaths:** |  |  | 1.40 | 0.206 |  |  | 0.00 | 1.000 |
| NO | 7 (58.3%) | 5 (41.7%) |  |  | 9 (75.0%) | 3 (25.0%) |  |  |
| YES | 16 (84.2%) | 3 (15.8%) |  |  | 13 (68.4%) | 6 (31.6%) |  |  |
| **Number of progressions:** |  |  | 1.13 | 0.185 |  |  | 3.39 | 0.032 |
| NO | 5 (55.6%) | 4 (44.4%) |  |  | 9 (100.0%) | 0 (0.0%) |  |  |
| YES | 18 (81.8%) | 4 (18.2%) |  |  | 13 (59.1%) | 9 (40.9%) |  |  |

^a^Chi-square test; ^b^Fisher's exact test; BVI, blood vessel invasion; HER-2, human epidermal growth factor receptor; PNI, perineural invasion;

**Table. S3** Efficacy outcomes of 5 patients with Extramammary Paget’s disease treated with RC48 at our center.

| **Patients** | **Age** | **T** | **N** | **Pre-treatment tumor markers** | **Post-treatment tumor markers** | **HER2 status of primary site** | **HER2 status of metastatic site** | **Location** | **Treatment** | **Cycles** | **Efficacy** | **Toxicity** |
| --- | --- | --- | --- | --- | --- | --- | --- | --- | --- | --- | --- | --- |
|  |  |  |  | **CEA (ng/ml)** | **CEA (ng/ml)** |  |  |  |  |  |  |  |
| **#1** | 75 | 1 | 2 | 108 | 23.90 | 3+ | 3+ | Retroperitoneal lymph nodes | Adjuvant therapy: Disitamab vedotin (Aidixi®) 2mg/kg iv drip every 2 weeks； | 3 | SD | G2 alopecia, G1 nausea |
| **#2** | 70 | 2 | 2 | 912 | 199 | 3+ | 3 | Pelvic lymph nodes | Adjuvant therapy: Disitamab vedotin (Aidixi®) 2mg/kg iv drip every 2 weeks； | 10 | PR | G1 anemia, G1 proteinuria, G2 alopecia, G1 peripheral neuropathy, G1 nausea, G1 rash |
| **#3** | 72 | 2 | 2 | 9.69 | 4.06 | 2+ | 2 | Pelvic lymph nodes | Adjuvant therapy: Disitamab vedotin (Aidixi®) 2mg/kg iv drip every 2 weeks； | 8 | PR | G1 anemia, G2 rash, G2 alopecia, G2 oral mucositis, G2 nausea |
| **#4** | 69 | 2 | 2 | 25.5 | 24.80 | 2+ | 2+ | Pelvic lymph nodes | Adjuvant therapy: Disitamab vedotin (Aidixi®) 2mg/kg iv drip every 2 weeks； | 6 | PR | G2 alopecia , G1 nausea, G1 anorexia. |
| **#5** | 63 | x | 2 | NA | NA | NA | 1+ | Pelvic lymph nodes | Adjuvant therapy: Disitamab vedotin (Aidixi®) 2mg/kg iv drip every 2 weeks； | 4 | PR | G1 anorexia, G2 peripheral neuropathy. G2 alopecia. |

CEA, carcinoembryonic antigen; HER2, human epidermal growth factor receptor; NE, not evaluated; OS, overall survival; PFS, progression-free survival; PR, partial response; SD, stable disease,

**Table. S4** Comparison of the current study and previous studies on the efficacy and safety of different regimens in patients with advanced EMPD.

|  | **Present study** | **Yoshino et al.** | **Kato et al.** | **Wada et al** | **Sohn et al** | **Hashimoto et al** |
| --- | --- | --- | --- | --- | --- | --- |
| **Study design** | Single-institute retrospective  study | Multiple-institute retrospective  study | Single-institute retrospective  study | Single-institute retrospective  study | Multiple-institute retrospective  study | Single-institute retrospective  study |
| **Administration dose** | RC48(2mg/kg, every 2 weeks) | DTX (60 mg/m^2^  , monthly) | DTX (60 mg/m^2^  , every 3 weeks) | DTX (60 mg/m^2^  , monthly) or S-1 (60 mg/m^2^ day 1-28, every 6 weeks) | platinum-based(Cisplatin/Carboplatin/ plus 5-FU)  or  taxane-based(PTX/DTX) chemotherapy | DTX (60–75 mg/m^2^, tri-weekly or monthly); DTX (40 mg/m^2^, day 1)+S-1(40 mg/m^2^, day 1–14)m, every 4 weeks; PTX  (80 mg/m^2^, weekly); 5-FU (500 mg/m^2^)+cisplatin (5mg/m^2^, five days/week); DTX (75 mg/m^2^) +trastuzumab (8mg/kg) as the first dose, followed by DTX(75 mg/m^2^)+trastuzumab (6mg/kg) |
| **Total patients** | 5 | 13 | 22 | 21 | 31 | 14 |
| **No. of confirmed cases** | 5 | 12 | 22 | 17+4 | 23+8 | 14 |
| **Mean age (range), years** | 70(63-75) | 71(62-83) | 74 (54–86) | 73(51-88)/78(72-80) | 64(47-76)/67(57-75) | 72(58-82) |
| **lymph node metastasis** | 5 | 9 | 14 | 17+4 | 23+8 | 14 |
| **Efficacy** | 4PR  1SD | 6PR  1SD  2PD | 7PR  6SD  1PD | 2CR,6PR,5SD,4PD/  0CR,0PR,1SD,3PD | 10PR,7SD,2PD,4NE/1CR,4PR,2SD,1NE | 7PR,2SD,5PD |
| **Objective response rate** | 80.0% | 66.7% | 50.0% | 47.1% or 0.0% | 43.5% or 62.5% | 50% |
| **Disease control rate** | 100.0% | 77.8% | 92.8% | 76.5% or 25.0% | 73.9% or 87.5% | 64.3% |
| **AE** | 5 (100.0%) | 13 (100.0%) | N/A | N/A | N/A | 10(71.4%) |
| **Hematological AE** | 4 (80.0%) | 13 (100.0%) | N/A | N/A | N/A | 10(71.4%) |
| Grade 3 to 5 hematological AE |  |  |  |  |  |  |
| Leukopenia | 0(0.0%) | 5 (38.5%) | N/A | N/A | N/A | 9(64.3%) |
| Neutropenia | 0(0.0%) | 8 (61.5%) | N/A | N/A | N/A | 10(71.4%) |
| Anemia | 0(0.0%) | 0(0.0%) | N/A | N/A | N/A | 2(14.3%) |
| Febrile neutropenia | 0(0.0%) | 4 (30.8%) | N/A | N/A | N/A | 3(21.4%) |
| Thrombocytopenia | 0(0.0%) | NA | N/A | N/A | N/A | 1(7.1%) |
| Grade 1 to 2 hematological AE |  |  |  |  |  |  |
| Leukopenia | 0(0.0%) | N/A | N/A | N/A | N/A | N/A |
| Neutropenia | 0(0.0%) | N/A | N/A | N/A | N/A | N/A |
| Anemia | 3 (60.0%) | N/A | N/A | N/A | N/A | N/A |
| Thrombocytopenia | 0(0.0%) | N/A | N/A | N/A | N/A | N/A |
| **Non-hematological AE（grade 1 to 2）** |  |  |  |  |  |  |
| Alopecia | 5 (100.0%) | N/A | N/A | N/A | N/A | N/A |
| Nausea | 4 (80.0%) | N/A | N/A | N/A | N/A | N/A |
| Anorexia | 2 (40.0%) | N/A | N/A | N/A | N/A | N/A |
| Peripheral neuropathy | 2 (40.0%) | N/A | N/A | N/A | N/A | N/A |
| Oral mucositis | 1 (20.0%) | N/A | N/A | N/A | N/A | N/A |
| Rash | 2 (40.0%) | N/A | N/A | N/A | N/A | N/A |

AE, adverse events; CR, complete response; DCR, disease control rate; DTX, docetaxel; EMPD, extramammary Paget’s disease; N/A, not applicable; NE, not evaluated; PD, progressive disease; PR, partial response; PTX, paclitaxel; RR, response rate; S-1, tegafur; SD, stable disease.
